# Dual ROCK1/2–MYLK4 Kinase Inhibition Preserves Visual Function in a Rat Model of Neuromyelitis Optica Spectrum Disorder Optic Neuritis

**DOI:** 10.3390/cells14211712

**Published:** 2025-10-31

**Authors:** Chin-Te Huang, Monir Hossen, Tu-Wen Chen, Chih-Wei Fu, Yi-Hsun Chen, Tzu-Lun Huang, Rong-Kung Tsai

**Affiliations:** 1 Institute of Eye Research, Hualien Tzu Chi Hospital, Buddhist Tzu Chi Medical Foundation, Hualien 970473, Taiwan; 2 Department of Ophthalmology, Chung Shan Medical University Hospital, Taichung 402306, Taiwan; 3 Department of Ophthalmology, School of Medicine, College of Medicine, Chung Shan Medical University, Taichung 402306, Taiwan; 4 Biomedical Technology and Device Research Laboratories, Industrial Technology Research Institute, Hsinchu 310401, Taiwan; 5 Department of Ophthalmology, Far Eastern Memorial Hospital, Banqiao District, New Taipei City 220216, Taiwan; 6 Department of Electrical Engineering, Yuan Ze University, Chung-Li, Taoyuan 320315, Taiwan; 7 Institute of Medical Sciences, Tzu Chi University, Hualien 970374, Taiwan; 8 Doctoral Degree Program in Translational Medicine, Tzu Chi University and Academia Sinica, Hualien 970374, Taiwan

**Keywords:** neuromyelitis optica, optic neuritis, retinal ganglion cells, neuroinflammation, protein kinase inhibitors

## Abstract

**Highlights:**

**What are the main findings?**

- Intravitreal administration of ITRI-E-(S)4046 (dual ROCK1/2–MYLK4 inhibitor) preserved visual-evoked potentials and retinal ganglion cell survival in an NMOSD rat optic neuritis model.
- TRI-ES suppressed optic nerve inflammation, demyelination, and glial activation while promoting anti-inflammatory (M2) macrophage polarization.

**What is the implication of the main finding?**

- Dual ROCK/MYLK4 inhibition represents a novel neuroprotective strategy that may complement immunotherapy in NMOSD-related optic neuritis.
- This study supports the translation of kinase-targeting therapeutics aimed at preserving visual function in autoimmune demyelinating diseases.

**Abstract:**

**Background**: Neuromyelitis optica spectrum disorder (NMOSD) causes severe optic nerve (ON) inflammation and vision loss. Current treatments remain limited, prompting exploration of new therapeutic strategies. This study evaluated the efficacy of ITRI-E-(S)4046 (ITRI-ES), a dual ROCK1/2 and MYLK4 kinase inhibitor, in a rat model of NMOSD optic neuritis. **Methods**: NMOSD-like optic neuritis was induced in rats by applying NMOSD patient serum-soaked sponges around the ON. Rats received intravitreal injections of either 0.2% ITRI-ES, phosphate-buffered saline (PBS), or intraperitoneal methylprednisolone (MP). Visual function was assessed using flash visual-evoked potentials (fVEP). Retinal ganglion cell (RGC) survival and apoptosis were quantified using FluoroGold retrograde labeling and TUNEL assay. ON inflammation and demyelination were evaluated via immunohistochemistry and Western blot analysis of aquaporin-4 (AQP4), myelin basic protein (MBP), glial fibrillary acidic protein (GFAP), and inflammatory markers. **Results**: ITRI-ES significantly preserved visual function, restoring fVEP amplitudes (~36 μV vs. ~21 μV in PBS-treated, *p* < 0.05) and RGC density (~85% of normal vs. ~37% PBS). RGC apoptosis was reduced (~2.3-fold lower vs. PBS, *p* < 0.05). PBS-treated rats showed decreased AQP4 and MBP (2.5–2.8-fold vs. sham) and increased GFAP (2.8-fold). ITRI-ES maintained higher AQP4 (~3.5-fold) and MBP (~1.5-fold) levels, suppressed GFAP (~5.5-fold vs. PBS), reduced NF-κB, IL-1β, TNF-α, microglia activation, and macrophage infiltration, and increased anti-inflammatory Arg1 and CD206 markers (~3-fold vs. PBS). **Conclusions**: ITRI-ES alleviates optic nerve inflammation, preserves retinal integrity, and maintains visual function in NMOSD-associated optic neuritis, underscoring kinase inhibition as a promising therapeutic strategy.

## 1. Introduction

Neuromyelitis optica spectrum disorder (NMOSD) is a severe autoimmune demyelinating condition characterized by inflammatory attacks on the optic nerves (ONs) and spinal cord. Although NMOSD is relatively rare (estimated 1–5 cases per 100,000), damage to the ON can lead to profound, often irreversible visual loss, while spinal cord lesions may cause paralysis [1,2]. NMOSD is most often associated with IgG autoantibodies against aquaporin-4 (AQP4), the predominant water channel in the central nervous system (CNS) [3,4]. Binding of anti-AQP4 autoantibodies to astrocytic AQP4 receptors initiates complement-mediated and cell-mediated cytotoxicity, resulting in astrocytes injury and AQP4 loss. The ensuing inflammatory cascade—including release of cytokines and immune cell recruitment—causes oligodendrocyte damage, secondary axonal degeneration, and neuronal death [5,6,7,8,9].

Despite advances in NMOSD diagnosis and immunopathogenesis, developing effective therapies to prevent vision loss remains a challenge. Significant ON damage during NMOSD attacks often results in permanent visual dysfunction [10]. While high-dose corticosteroids and plasma exchange are standard acute treatments, they do not always prevent ON damage [11,12,13,14]. Long-term immunosuppressants (e.g., rituximab, mycophenolate, azathioprine) help reduce relapse frequency, and since 2019 three monoclonal antibody therapies (eculizumab, satralizumab, inebilizumab) have been approved for AQP4-IgG^+^ NMOSD [15,16]. These targeted biologics can dramatically lower relapse risk—eculizumab, for example, reduces relapses by ~94% [17]—yet even with these advances, patients may still suffer irreversible ON damage and residual visual loss. There remains a pressing need for adjunctive neuroprotective treatments to mitigate ON injury during NMOSD attacks.

Another hurdle in NMOSD research has been the lack of an ideal animal model replicating optic neuritis. Traditional experimental models, including passive transfer of AQP4-IgG with complement, AQP4-specific T cells, or experimental autoimmune encephalomyelitis (EAE)-based models, provide insights but failed to fully recapitulate human NMOSD ON pathology [18]. Many models require complex immunization or direct CNS injections and often introduce confounding inflammation or risk mechanical ON damage [19]. In this study, we employed a simplified rat model of NMOSD optic neuritis induced by placing an NMO patient’s serum-soaked sponge around the ON. This localized delivery of pathogenic antibodies provoked ON inflammation, AQP4 loss, and demyelination without the need for invasive intra-ON injections, closely mirroring the astrocytopathy and optic nerve localization of human NMOSD lesions.

Rho-associated protein kinase (ROCK) has been implicated in neuroinflammatory and neurodegenerative processes, including ocular disorders [20]. ROCK inhibitors have emerged as potential therapies in ophthalmology, with proven benefits in conditions such as glaucoma, corneal injury, retinal ischemia, and optic neuropathies [21,22]. In the CNS, ROCK signaling promotes inflammatory cytokine production and macrophage/microglial activation, while inhibiting axonal growth and promote neuronal survival [23,24]. Accordingly, ROCK inhibition can shift macrophage toward an anti-inflammatory (M2) phenotype, suppress microglia activation, and support axon regeneration [25,26].

ITRI-E-(S)4046 (ITRI-ES) is a novel dual kinase inhibitor of ROCK1/2 and myosin light-chain kinase family member 4 (MYLK4), developed by the Industrial Technology Research Institute (ITRI) in Taiwan [27]. Originally designed to lower intraocular pressure (IOP) in glaucoma, ITRI-ES showed superior efficacy in preclinical glaucoma models [28]. Unlike conventional ROCK inhibitors, ITRI-ES concurrently blocks ROCK and MYLK4, two complementary regulators of cytoskeletal contractility. In neuroinflammatory contexts, ROCK and MLCK independently promote blood–brain barrier disruption, immune cell infiltration, and astrocytic injury through myosin light-chain (MLC) phosphorylation and actomyosin contraction [29]. Selective ROCK inhibition alone has shown protective efficacy in demyelinating disease models such as experimental autoimmune encephalomyelitis (EAE), where fasudil reduced disease severity, T-cell proliferation, and inflammatory cytokine production [30]. Similarly, MLCK inhibition enhances endothelial barrier integrity under inflammatory stress [29]. Since these pathways act synergistically to exacerbate inflammation and barrier leakage, their combined inhibition is expected to yield additive or even synergistic benefits. Thus, we hypothesized that dual ROCK/MYLK4 blockade with ITRI-ES would provide superior neuroprotection in NMOSD-related optic neuritis by simultaneously suppressing converging mechanisms of barrier breakdown and immune activation, beyond what single-pathway inhibition could achieve.

In this study, we evaluated the therapeutic potential of intravitreal 0.2% ITRI-ES in a rat NMOSD optic neuritis model, in comparison to the standard corticosteroid treatment, methylprednisolone (MP). We assessed visual function, RGC survival, and neuroinflammation to determine whether dual ROCK/MYLK4 kinase inhibition can preserve retinal function and attenuate ON damage in NMOSD.

## 2. Materials and Methods

### 2.1. Animal Model and Study Design

**Figure 1:**
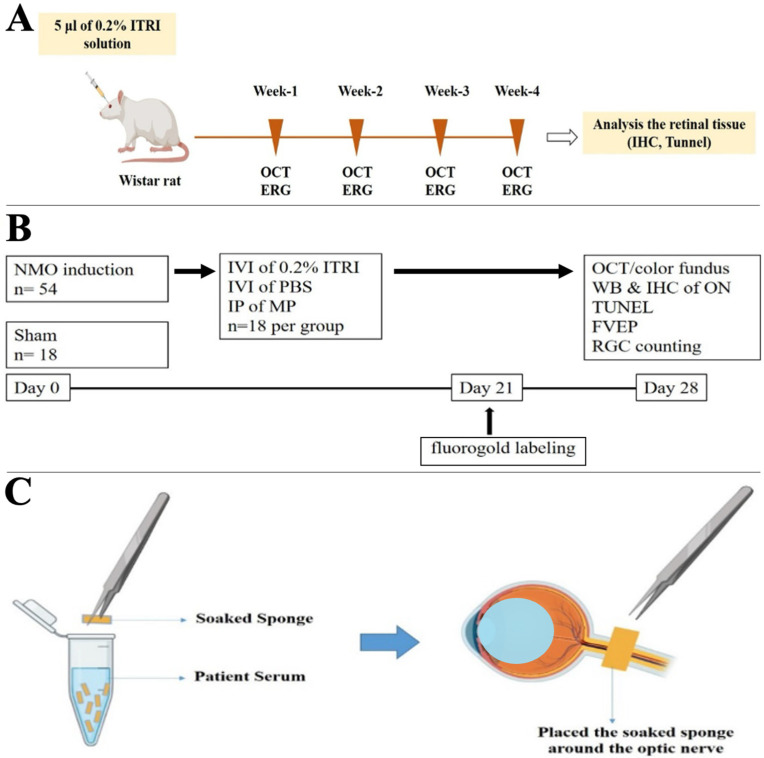
Experimental design for evaluating 0.2% ITRI-E-(S)4046 solution (ITRI-ES) safety and therapeutic efficacy in neuromyelitis optica spectrum disorder (NMOSD) rats. (**A**) Timeline of safety evaluation in healthy rats following intravitreal injection (IVI) of 5 µL ITRI-ES. Structural and functional assessments, including optical coherence tomography (OCT) and electroretinography (ERG), were performed at weeks 1, 2, 3, and 4 post-injection. At week 4, retinal tissues were collected for immunohistochemistry (IHC) and Terminal deoxynucleotidyl transferase dUTP nick end labeling (TUNEL) analysis. (**B**) Experimental protocol for NMOSD model induction and treatment. NMOSD was induced in 54 rats, with 18 rats serving as sham controls. On day 0, animals received IVI of ITRI-ES, phosphate-buffered saline (PBS), or intraperitoneal injection (IP) of methylprednisolone (MP). Fluorogold labeling was performed on day 21. On day 28, evaluations included OCT, fundus imaging, Western blot (WB) and IHC of the optic nerve (ON), TUNEL assay, flash visual evoked potentials (fVEP), and retinal ganglion cell (RGC) counting. (**C**) Schematic diagram of the NMO model induction method. A sterile sponge soaked in patient serum was applied around the ON to deliver human anti-AQP4 antibodies, mimicking NMOSD pathology.

Adult male Wistar rats (6 weeks old, 125–250 g) were housed under standard conditions with food and water ad libitum. Two experimental paradigms were conducted. In the first, a safety evaluation, six normal rats received a 5 μL intravitreal injection of 0.2% ITRI-ES in each eye to assess ocular tolerance. In the second experiment, a total of 72 rats were used to induce NMOSD-like optic neuritis and test treatments (Figure 1A,B). Rats were randomly assigned (n = 18 per group) to: sham, undergoing ON surgery with a saline-soaked sponge (no patient serum); PBS (phosphate-buffered saline), NMOSD induction (patient serum) followed by 5 µL intravitreal PBS; MP, NMOSD induction followed by systemic methylprednisolone (30 mg/kg intraperitoneally once daily for 3 days); and ITRI-ES, NMOSD induction followed by 5 µL intravitreal 0.2% ITRI-ES (Figure 1B). In all treatment groups, the intervention was administered immediately after the ON was exposed to the serum-soaked sponge (day 0).

### 2.2. NMOSD Induction Method

Briefly, under anesthesia (ketamine 100 mg/kg and xylazine 10 mg/kg, intramuscular) and after topical analgesia, a lateral canthotomy was performed to access the ON. A small sterile sponge (5 × 1.5 mm) soaked in AQP4-IgG seropositive NMOSD patient serum (titer 1:320) was gently wrapped around the exposed anterior ON (Figure 1C). The conjunctival incision was then sutured and antibiotic ointment applied. Sham-operated rats had an identical procedure with a saline-soaked sponge. Rats were monitored daily for well-being and any ocular complications until study end (28 days). The human serum used was obtained with informed consent, and its anti-AQP4 antibody titer was confirmed by enzyme-linked immunosorbent assay (ELISA).

In contrast to the method described by Matsumoto et al., where the optic nerve sheath was surgically removed to allow direct exposure to AQP4+ patient serum [19], we developed a modified approach that preserved the optic nerve sheath. This avoids artificial mechanical injury and better reflects the natural disease course of NMOSD. Compared with the model by Asavapanumas and Ratelade et al., which required continuous intracranial infusion near the optic chiasm to induce robust pathology [4], our method provides a technically simpler yet reproducible means of inducing optic neuritis using patient serum.

### 2.3. ITRI-ES Preparation and Administration

ITRI-E-(S)4046 (API batch: 20200604, ITRI, Hsinchu, Taiwan) is an (S)-N-(4-(1H-pyrazol-4-yl)phenyl)-3-amino-2-phenylpropanamide dihydrochloride, 4-carboxamide-phenyl substituted phenyl pyrazole-based small-molecule ROCK/MYLK4 inhibitor designed through fragment-based approaches as well as molecular modeling methods. The target compound was synthesized via propylphosphonic anhydride (T3P)-mediated coupling with tert-butyl 4-(4-aminophenyl)-1H-pyrazole-1-carboxylate and (S)-3-(1,3-dioxoisoindolin-2-yl)-2-phenylpropanoic acid to obtain intermediate of tert-butyl (S)-4-(4-(3-(1,3-dioxoisoindolin-2-yl)-2-phenylpropanamido)phenyl)-1H-pyrazole-1-carboxylate, followed by N-tert-butyloxycarbonyl and N-phthaloyl deprotection to obtain free-form amine of (S)-N-(4-(1H-pyrazol-4-yl)phenyl)-3-amino-2-phenylpropanamide, and final amine dihydrochloride salts formation with 12 M HCl in dichloromethane and isopropyl alcohol.

For dosing, the compound was formulated as a sterile 0.2% (2 mg/mL) solution in polyethylene glycol-400 (PEG, Cat. 8117, Fluka Chemie AG, Buchs, Switzerland) solution and 10% D-mannitol (Cat. M1902, Sigma-Aldrich, St. Louis, MO, USA) in 40 mM boric acid buffer (Cat. 0084-01, J.T. Baker/Avantor, Radnor, PA, USA). The pH was adjusted with 1.0 M sodium hydroxide standard solution (Cat. VW3222-1, VWR International, West Chester, PA, USA) to pH 5 ± 0.1. A 5 μL volume of ITRI-ES solution (or PBS for controls) was injected intravitreally using a microsyringe at the time of NMOSD induction. The dose was selected based on preliminary studies and is equivalent to 10 μg delivered per eye. Systemic MP was administered at 30 mg/kg IP (approximately equivalent to a high clinical dose) starting on day 0, repeated for three consecutive days, to model acute attack treatment.

### 2.4. Outcome Measures

Animals were evaluated 28 days after induction for structural, functional, and molecular outcomes. In the safety cohort, serial examinations were also performed weekly. Fundus photography and optical coherence tomography (OCT) were used to monitor retinal structure and ON head (ONH) morphology using the Micron IV retinal microscope (Phoenix Research Labs, Pleasanton, CA, USA). The measurement of ONH width, the width of Bruch’s membrane opening (BMO), at the midpoint of the ON was conducted through a linear scan. In contrast, retinal thickness was determined via a circular scan around the ON. In the safety study, OCT scans at baseline and weeks 1, 2, 3, and 4 post-injection were analyzed for retinal thickness and optic nerve head width, to detect any ITRI-ES-induced changes.

Full-field electroretinography (ERG, Colordome Ganzfield, Diagnosys LLC, Lowell, MA, USA) was performed under scotopic and photopic conditions to assess retinal function, with a- and b-wave amplitudes measured weekly for 4 weeks after injection. At day 28, flash visual-evoked potentials (fVEPs) were recorded using a Celeris system (Diagnosys LLC, Lowell, MA, USA) to evaluate visual pathway function. Under anesthesia, transcranial electrodes were placed over the primary visual cortex and reference sites as described previously in our lab’s study [2,31]. fVEPs responses to strobe flash stimuli (30 cd·s/m^2^, 1.02 Hz) were averaged, and the P1–N2 amplitude was measured for each eye.

RGC survival was quantified by retrograde labeling according to our previous study [2,31]. One week before sacrifice (day 21), 5% FluoroGold (2 μL) was stereotactically injected into both superior colliculus to label RGCs projecting from each eye. On day 28, rats were euthanized. Eyes and optic nerves were harvested and fixed in 4% paraformaldehyde. Retinas were dissected as whole-mounts and examined by fluorescence microscopy (Axio Scope A1, Zeiss, Oberkochen, Germany) for FluoroGold-labeled RGCs. RGC densities were calculated in four central retinal fields (~1 mm from the ONH) and averaged per eye.

Apoptotic RGCs were assessed using a terminal deoxynucleotidyl transferase dUTP nick-end labeling (TUNEL) assay (DeadEndTM Fluorometric TUNEL System, Promega Corporation, Madison, WI, USA) on retinal cryosections. TUNEL-positive cells in the ganglion cell layer were counted in six sections per eye and averaged.

ON pathology and inflammation were analyzed by immunohistochemistry (IHC) and Western blot. Tissue sections underwent antigen retrieval and blocking, then were incubated with primary antibodies against ionized calcium binding adaptor molecule 1 (Iba1 for microglia, diluted 1:100; Abcam, San Francisco, CA, USA), and CD68 (ED1 for macrophages, diluted 1:100; MCA341R; Bio-Rad, Hercules, CA, USA). Appropriate secondary antibodies were applied to the tissue samples for one hour at a concentration of 1:400 in PBST. As a final step, the tissue samples were mounted using FluoroshieldTM with 4′,6-diamidino-2-phenylindole (DAPI) and counterstained with DAPI. Fluorescent images were captured by fluorescence microscope equipped with appropriate filters.

For Western blot, ON tissue (two ON samples pooled per group) was homogenized in RIPA buffer with protease/phosphatase inhibitors (Cat: 78442, Invitrogen, Waltham, MA, USA). Equal amounts of protein (20 μg) were separated by SDS-PAGE and transferred to polyvinylidene difluoride (PVDF) membranes. Blots were probed overnight at 4 °C with primary antibodies including NFκB (1:1000, ab16502, Abcam, Cambridge, UK), Iba1 (1:1000, ab178680, Abcam, Cambridge, UK), interleukin-1 Beta (IL-1β, 1:1000; AF-401-NA, R&D system, Minneapolis, MN, USA), GFAP (1:1000 ab68428, Abcam, Cambridge, UK), AQP-4 (1:1000, MA5-24587, Thermo Fisher Scientific, Waltham, MA, USA), arginase-1 (Arg1, 1:1000, ab91279, Abcam, Cambridge, UK), CD206 (mannose receptor, 1:500, ab64693, Abcam, Cambridge, UK), myelin basic protein (MBP) (1:1000, ab24567, Abcam, Cambridge, UK), tumor necrosis factor alpha (TNF-α, 1:1000, ab6671, Abcam, Cambridge, UK), and β-actin (1:5000, A1978, Sigma-Aldrich, St. Louis, MO, USA). After horseradish peroxidase (HRP)-conjugated secondary antibodies (1:10,000, Bio-Rad, Hercules, CA, USA) for one hour at room temperature, bands were visualized by chemiluminescence (ECL) imaging systems (Analytik Jena, Jena, Germany) and ChemiDoc MP Imaging Systems (Bio-Rad, Hercules, CA, USA), and quantified using iBright Analysis software (Invitrogen, Carlsbad, CA, USA).

### 2.5. Statistical Analysis

Data are presented as mean values with standard deviations (SD). Group comparisons were made using the Kruskal–Wallis test with appropriate post hoc analysis (nonparametric ANOVA, given sample sizes and data distributions). A *p*-value < 0.05 was considered statistically significant.

### 2.6. Standard Protocol Approvals, Registrations, and Patient Consent

All animal procedures were approved by the Institutional Animal Care and Use Committee (IACUC) of Hualien Tzu Chi Medical Center (IACUC Approval No. 110-43) and adhered to the Association for Research in Vision and Ophthalmology (ARVO) Statement for the Use of Animals in Ophthalmic and Vision Research. The human serum used was obtained with informed consent under an IRB-approved protocol from the Research Ethics Review Committee of Far Eastern Memorial Hospital (Approval No. 106168-E).

## 3. Results

### 3.1. Safety of Intravitreal ITRI-ES

**Figure 2:**
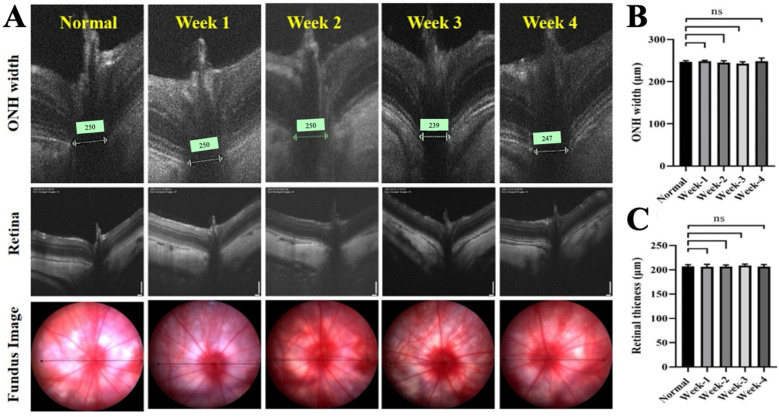
Retinal structural assessment following intravitreal 0.2% ITRI-E-(S)4046 solution (ITRI-ES) injection. (**A**) Representative fundus and optical coherence tomography (OCT) images showing retinal structure and optic nerve head (ONH) at baseline and weeks 1 to 4 after ITRI-ES injection. (**B**) Quantification of ONH width across time points revealed no significant changes compared to baseline. (**C**) Quantification of retinal thickness showed consistent values throughout the 4-week period post-injection. Data are presented as mean ± SD (n = 6); ns = not significant.

**Figure 3:**
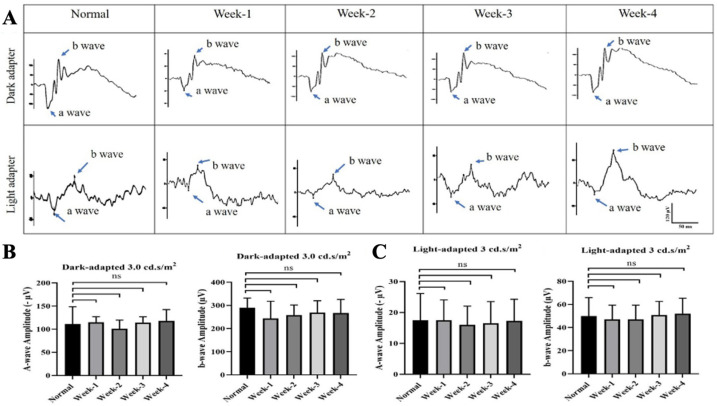
Functional evaluation of retinal response via electroretinography (ERG). (**A**) Dark-adapted ERG responses in eyes injected with 0.2% ITRI-E-(S)4046 solution (ITRI-ES) at weeks 1, 2, 3, and 4, showing no significant changes in a-wave and b-wave amplitudes compared to normal controls. Light-adapted ERG responses at the same time points, also showing no significant changes in retinal function. (**B**) Quantitative analysis of a-wave and b-wave amplitudes in dark-adapted conditions. (**C**) Quantitative analysis of a-wave and b-wave amplitudes in light-adapted conditions. ERG responses indicate that the ITRI-ES did not adversely affect retinal function compared to normal controls. Data are presented as mean ± SD, n = 6 per group, ns = not significant.

No adverse clinical signs were observed in rats following intravitreal ITRI-ES injection. Fundus examination and OCT imaging revealed no retinal pathology or ON head swelling through 4 weeks post-injection. Retinal thickness and ON head width in ITRI-ES-treated eyes remained comparable to baseline and control eyes (all changes <3% from baseline, *p* > 0.05). Representative OCT images confirmed no structural alterations (Figure 2A). Likewise, ERG recordings showed that ITRI-ES did not affect retinal function. Scotopic a-wave and b-wave amplitudes at 1–4 weeks post-injection were unchanged from pre-injection values and similar to untreated eyes (e.g., week 4 scotopic b-wave 267 ± 58.9 μV vs. 290 ± 41.5 μV normal, *p* > 0.5) (Figure 3). Photopic ERGs also remained normal.

**Figure 4:**
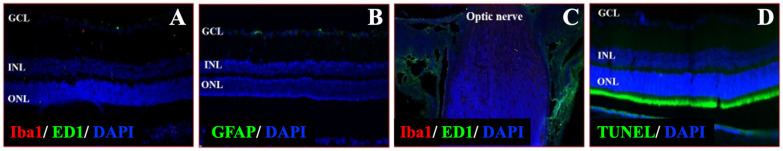
Molecular and cellular resilience in the retina and optic nerve post dual kinase inhibitor intravitreal injections. (**A**–**C**) Immunohistochemistry (IHC) staining for Iba1, ED1, and GFAP in the retina and optic nerve. No positive signals were observed in IHC for Iba1, ED1, and GFAP, indicating no inflammation or glial activation in both the retina and the optic nerve. (**D**) TUNEL assay for detecting apoptosis in the retinal ganglion cell (RGC) layer. The TUNEL assay revealed no apoptotic cells in the retinal ganglion cell (RGC) layer. The 0.2% ITRI-E-(S)4046 solution (ITRI-ES) did not induce significant glial activation or apoptosis in the retina compared to normal controls, supporting the safety of the ITRI-ES. Data are presented as mean ± SD, n = 6 per group. (GCL, ganglion cell layer; INL, inner nuclear layer; ns, not significant; ONL, outer nuclear layer).

Histologically, ITRI-ES-treated eyes showed no signs of inflammation or gliosis. IHC for Iba1 (microglia), ED1 (macrophages), and GFAP (astrocytes) was negative, indistinguishable from controls (Figure 4A–C). No TUNEL-positive cells were detected in RGC layers (Figure 4D). These safety results indicate that intravitreal 0.2% ITRI-ES is well tolerated, causing no significant retinal toxicity or functional impairment over at least one month.

### 3.2. ITRI-ES Preserves Visual Function and RGCs in NMOSD Optic Neuritis

**Figure 5:**
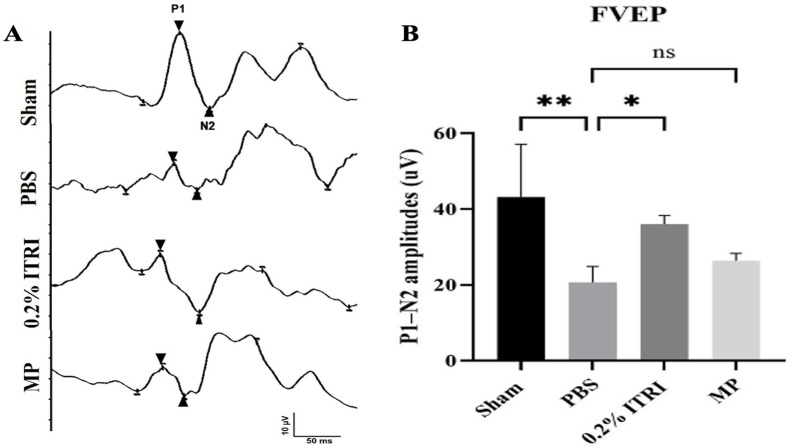
Preservation of visual function in the NMOSD model. (**A**) Flash visual evoked potential (fVEP) responses on day 28 post-NMOSD induction. (**B**) Quantitative analysis of P1–N2 amplitudes in the sham, PBS-treated, 0.2% ITRI-E-(S)4046 solution (ITRI-ES)-treated, and MP-treated groups. Treatment with ITRI-ES preserved visual function significantly compared to the PBS-treated group. Data are presented as mean ± SD, n = 6 per group. (*: *p* < 0.05, **: *p* < 0.01; MP, methylprednisolone; ns, not significant; PBS, phosphate-buffered saline).

At 28 days after NMOSD induction, the PBS-treated rats exhibited dramatically impaired visual function. The flash VEP P1–N2 amplitude in PBS eyes (20.7 ± 4.2 μV) was reduced by ~58% compared to sham (49.3 ± 17.4 μV, *p* < 0.01), confirming severe ON conduction deficit (Figure 5). In contrast, ITRI-ES-treated eyes showed a significantly higher P1–N2 amplitude of 36.1 ± 2.2 μV. This represented a ~75% preservation of the normal VEP signal, and was significantly greater than the PBS group (*p* < 0.05). Notably, ITRI-ES also outperformed systemic MP in this model: the MP-treated group’s VEP amplitude (26.9 ± 1.6 μV) was only marginally better than PBS and did not reach statistical significance vs. PBS (*p* > 0.05), whereas ITRI-ES -treated eyes had clearly superior responses (*p* < 0.05 vs. MP). Previous studies have reported that glucocorticoids partially suppress inflammation in NMO models but fail to prevent irreversible axonal damage [8]. This aligns with our observation that methylprednisolone did not produce statistically significant protection compared with PBS. These findings indicate that a single intravitreal dose of ITRI-ES provided considerable protection of visual pathway function.

**Figure 6:**
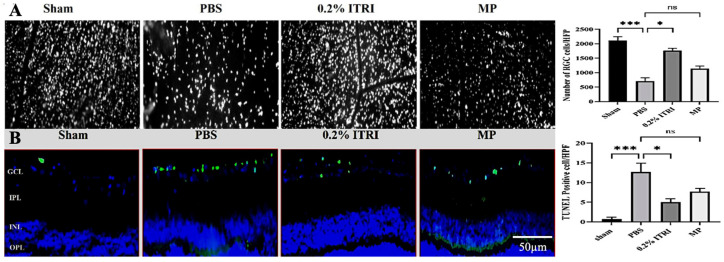
ITRI-ES preserves retinal ganglion cell survival and reduces apoptosis in NMOSD rats. (**A**) Fluoro-Gold retrograde labeling was used to visualize RGCs at day 28 post-NMOSD induction. The number of labeled RGCs in the PBS-treated group was significantly reduced compared to the sham group, whereas treatment with 0.2% ITRI-E-(S)4046 solution (ITRI-ES) or methylprednisolone (MP) increased RGC density. (**B**) TUNEL staining showed a marked increase in apoptotic RGCs in the PBS group, which was significantly reduced in the ITRI-ES-treated group. Quantitative analysis confirms the neuroprotective effect of ITRI-ES in promoting RGC survival and reducing apoptosis. (Scale bar = 200 μm in (**A**), 50 μm in (**B**); n = 6 per group; * *p* < 0.05; *** *p* < 0.001; ns, not significant).

Consistent with the functional benefits, ITRI-ES markedly protected RGC from NMOSD-related damage (Figure 6A). Retrograde RGC labeling and counts on day 28 revealed that PBS-treated eyes lost over half their RGCs, with RGC density falling to 784.7 ± 64.9 cells/mm^2^ compared to 2113 ± 144.6 cells/mm^2^ in sham controls (a 63% reduction, *p* < 0.001). Treatment with ITRI-ES rescued a large fraction of RGCs: the ITRI-ES group had 1794 ± 58.29 cells/mm^2^, which was 2.3-fold higher than the PBS group (*p* < 0.02) and not significantly different from the sham group. In contrast, systemic MP yielded only a partial RGC preservation (1138 ± 104.7 cells/mm^2^), which did not significantly differ from PBS (*p* = 0.844). Thus, ITRI-ES treatment prevented the majority of RGC death caused by the NMOSD insult, whereas the MP regimen was largely ineffective in saving RGCs.

In agreement with the RGC counts, TUNEL assays demonstrated significantly fewer apoptotic cells in ITRI-ES-treated retinas (Figure 6B). PBS-treated eyes averaged 13.3 ± 1.52 TUNEL+ cells per high-power field (HPF) in the ganglion cell layer, nearly 20-fold higher than sham (0.67 ± 0.58 cells/HPF, *p* < 0.001). ITRI-ES reduced this apoptotic count to 5.67 ± 0.58 cells/HPF (*p* < 0.02 vs. PBS), a 2.3-fold improvement, whereas MP-treated eyes showed 7.3 ± 0.58 cells/HPF (only a 1.8-fold reduction, *p* = 0.84 vs. PBS). These results confirm that ITRI-ES not only improves electrophysiological outcomes, but also significantly enhances RGC survival and reduces cell death in NMOSD optic neuritis.

### 3.3. ITRI-ES Mitigates Optic Nerve Demyelination and Astrocyte Injury

**Figure 7:**
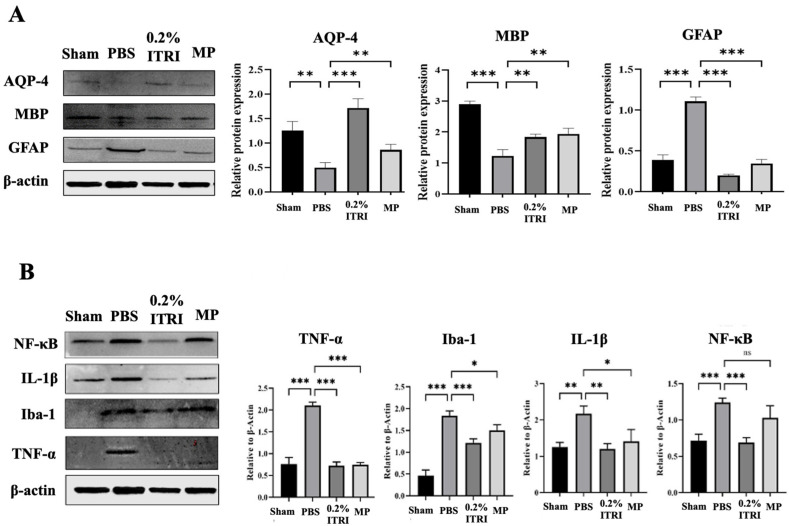
ITRI-ES treatment mitigates optic nerve pathology and inflammatory cytokine expression. (**A**) Western blot analysis showed decreased expression of AQP-4 and MBP and increased GFAP in the PBS-treated group, indicating NMOSD pathology. These alterations were significantly restored by ITRI-ES or MP. (**B**) Inflammatory cytokines (NF-κB, IL-1β, TNF-α) and microglial marker Iba1 were upregulated in the PBS group. Treatment with ITRI-ES significantly suppressed these inflammatory markers, indicating anti-inflammatory effects. Quantifications are normalized to β-actin. Data are presented as mean ± SD, n = 6 per group; * *p* < 0.05; ** *p* < 0.01; *** *p* < 0.001; ns, not significant.

The key pathological features of human NMOSD lesions include loss of AQP4, and myelin basic protein (MBP) [32]. Next, we examined molecular and histopathological markers in the ON to evaluate how ITRI-ES affected NMOSD lesion pathology. In untreated NMOSD (PBS group), optic nerve sections showed classical features of NMOSD damage: loss of AQP-4 expression on astrocytes, loss of MBP and reactive astrogliosis with upregulated GFAP (Figure 7A). Relative to sham nerves, PBS-treated nerves had AQP4 levels reduced to ~40% (a 2.52-fold decrease, *p* = 0.001) and MBP to ~35% (2.83-fold decrease, *p* < 0.001). Moreover, in response to damage and inflammation in NMOSD lesions, astrocytes become reactive and hypertrophic, leading to increased expression of GFAP [33]. This elevated GFAP expression serves as a marker indicating that astrocytes are actively responding to the pathological changes in NMOSD [33]. GFAP immunoreactivity, conversely, was strongly increased (2.83-fold higher than sham, *p* < 0.001), reflecting extensive astrocyte activation. Treatment with ITRI-ES dramatically preserved these structural markers. ONs from the ITRI-ES group showed intense AQP4 and MBP staining similar to shams, whereas PBS-treated ONs were nearly devoid of AQP4. Quantitatively, ITRI-ES-treated nerves had AQP-4 levels approximately 3.46-fold higher than PBS (*p* = 0.001) and MBP 1.49-fold higher (*p* = 0.006). GFAP levels were correspondingly lower in treated eyes—ITRI-ES reduced GFAP by 5.52-fold compared to PBS (*p* < 0.001), indicating a near-complete suppression of the astrocyte hypertrophy/activation seen in untreated lesions. The MP-treated group also showed some preservation of AQP4 (1.73-fold, *p* < 0.001) and MBP (1.57-fold, *p* = 0.002) compared to PBS, and a significant decrease in GFAP (3.19-fold, *p* < 0.001). However, ITRI-ES was more effective than MP on all three markers, aligning with its superior RGC protection. These data suggest that ITRI-ES prevented much of the astrocytic damage and demyelination that characterize NMOSD ON lesions.

### 3.4. ITRI-ES Suppresses Neuroinflammation and Promotes an M2 Phenotype

NMOSD pathology is driven by immune-mediated mechanisms, including astrocyte–microglia interactions, pro-inflammatory cytokines, and complement activation. Previous research described that activated astrocytes respond to the presence of autoantibodies by releasing pro-inflammatory cytokines, including IL-1β and TNF-α. These cytokines can directly stimulate NF-κB activation within astrocytes. These cytokines are potent mediators of inflammation and can further activate microglia and recruit immune cells to the site of inflammation in the ON [34,35]. We evaluated key inflammatory mediators in the optic nerve to determine whether ITRI-ES modulated the lesion immune environment. In PBS-treated ONs, expression of NF-κB, IL-1β, and TNF-α was markedly upregulated (1.73–2.9-fold increase vs. sham by densitometry) consistent with robust inflammatory signaling (Figure 7B). Treatment with ITRI-ES significantly blunted this inflammatory response. Western blot showed that ITRI-ES reduced NF-κB and IL-1β levels by 1.73–1.8-fold compared to PBS (*p* < 0.005 each). TNF-α was also markedly lower (ITRI-ES group 35% of PBS, *p* < 0.001). Iba1, a microglial marker, was also elevated 3.94-fold (*p* < 0.001) in PBS ONs vs. sham, reflecting substantial microglial activation. MP treatment partially lowered pro-inflammatory responses (NF-κB, IL-1β, Iba1 levels reduced by 1.2–1.5-fold vs. PBS), but generally to a lesser degree than ITRI-ES, and some differences (e.g., NF-κB) did not reach significance for MP. Notably, ITRI-ES not only dampened pro-inflammatory factors but also appeared to promote an anti-inflammatory milieu.

**Figure 8:**
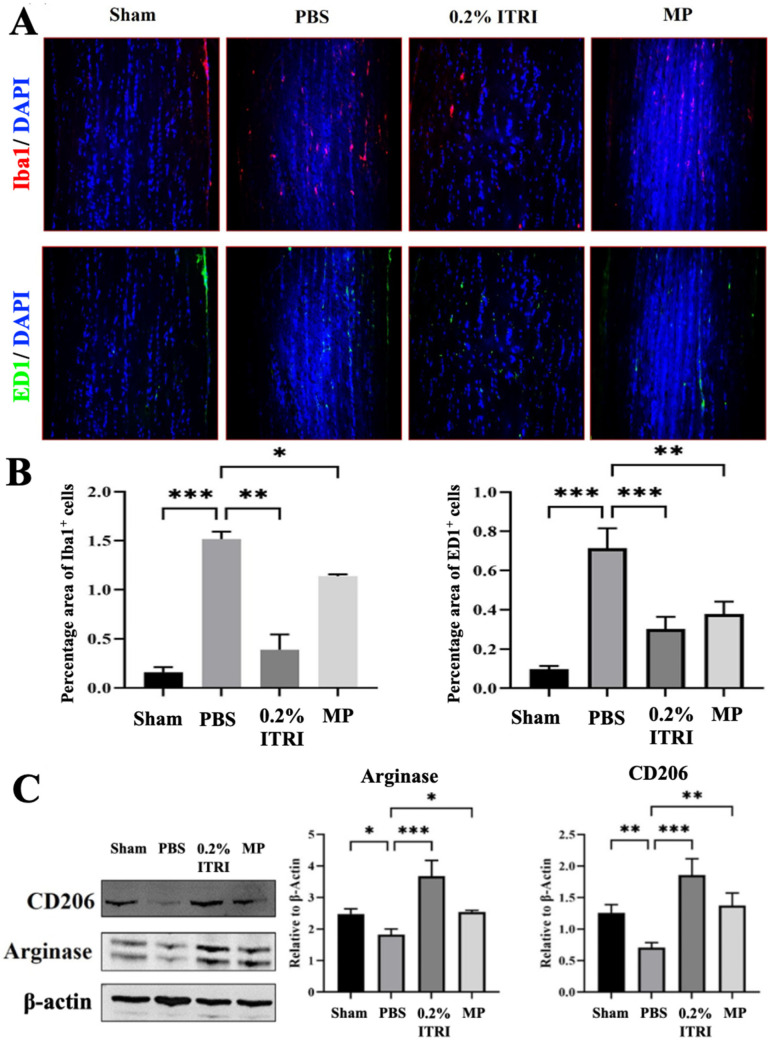
ITRI-ES inhibits microglial/macrophage activation and promotes M2 polarization in the optic nerve. (**A**) Representative immunofluorescence staining for Iba1 and ED1 showed increased microglial activation and macrophage infiltration in the PBS-treated group, which were reduced after ITRI-ES or MP treatment. (**B**) Quantitative analysis confirmed significantly lower percentages of Iba1^+^ and ED1+ cells in the ITRI-ES-treated group. (**C**) Western blot analysis of M2 polarization markers (CD206 and Arginase 1) demonstrated that ITRI-ES significantly enhanced M2 marker expression, indicating a favorable shift toward neuroprotective inflammation modulation. Data are shown as mean ± SD, n = 6 per group; * *p* < 0.05; ** *p* < 0.01; *** *p* < 0.001.

Microglial activation and macrophage infiltration were also observed in the NMOSD pathology. AQP4-IgG binding to astrocytes triggers a chain reaction. It activates astrocytes, leading to the production of cytokines, which then activate nearby microglia. This interplay between astrocytes and microglia amplifies CNS inflammation, making microglia significant contributors to the inflammatory response in NMOSD [4,36,37]. Furthermore, macrophage infiltration is closely linked to complement-dependent cytotoxicity mediated by membrane attack complexes (MACs) and the occurrence of localized inflammation in the ON during NMOSD [36]. IHC confirmed dense Iba1+ microglia and ED1+ macrophages infiltrating PBS-treated optic nerves, whereas sham nerves had few (Figure 8A). ITRI-ES-treated ONs had far fewer activated microglia and infiltrating macrophages. The number of Iba1+ cells in ITRI-ES-treated ONs was only 25% of that in PBS (a 3.89-fold reduction, *p* < 0.005). ED1+ macrophages were reduced by 2.29-fold vs. PBS.

We observed increased expression of M2 polarization markers Arg1 and CD206 in ITRI-ES-treated ONs (Figure 8B). Densitometry indicated Arg1 and CD206 protein levels were 3.1-fold and 3.2-fold higher in ITRI-ES vs. PBS groups, respectively (*p* < 0.001). In contrast, PBS and MP groups showed minimal expression of these M2 markers. This shift suggests that ITRI-ES not only suppresses deleterious M1-type inflammation but also fosters a more regenerative, M2-type immune response in the ON.

### 3.5. Validation of NMOSD Pathological Features in the Animal Model

The PBS-treated group exhibited significant visual impairment demonstrated by reduced fVEP P1–N2 amplitude (Figure 5), decreased RGC count (Figure 6A), increased retinal apoptosis (Figure 6B), loss of ON AQP-4 and MBP expression (Figure 7A), enhanced GFAP levels indicative of reactive gliosis (Figure 7A), and upregulated pro-inflammatory cytokines NF-κB, IL-1β, TNF-α (Figure 7B). Furthermore, ON sections revealed marked microglial activation and macrophage infiltration (Figure 8A), without significant polarization towards an anti-inflammatory M2 phenotype (Figure 8B). Through these outcomes, our rat model closely replicated human NMOSD ON pathology.

## 4. Discussion

We established an improved experimental model of NMOSD optic neuritis and found that targeting ROCK/MYLK4 signaling confers significant neuroprotection [4,18,19]. The novel rat model using a patient serum-soaked ON sponge reliably produced localized optic neuritis characterized by AQP4 loss, astrocyte activation, demyelination, and RGC degeneration, closely mimicking human NMOSD lesions [5]. This approach offers practical advantages over earlier models that required precise intrathecal or intra-ON injections [19,38,39]. The rat optic nerve is extremely delicate (0.1–0.2 mm diameter), making direct injection technically challenging and prone to off-target damage [40]. By instead delivering pathogenic antibodies via an external sponge, our model minimizes mechanical trauma while still allowing antibody and complement diffusion into the ON sheath. We observed that sham-operated rats (sponge with saline) showed no ON inflammation, confirming that the pathology in experimental rats was specifically due to NMOSD serum factors. This simplified, reproducible model should be useful for future translational studies of optic neuritis in NMOSD.

Our findings also provide insight into NMOSD disease mechanisms. In the PBS-treated rats, we noted a substantial reduction in AQP4 in the ON, but no outright loss of astrocyte by day 28. This aligns with reports of “sub-lytic” astrocyte injury in some NMOSD lesions, where AQP4 is lost without complete astrocyte destruction [32]. One explanation is that the complement levels delivered via patient serum were sufficient to damage astrocyte end-feet (causing AQP4 internalization or loss) but not enough to kill astrocytes outright. Prior studies have shown that injecting AQP4-IgG alone does not cause lesions, whereas co-injection with human complement does [38]. Another study also demonstrated that passive transfer of NMOSD-IgG with human complement near the optic chiasm in mice induced ON lesions with rapid loss of astrocytes [4,32]. In our model, the patient serum likely contained complement, but perhaps at a lower concentration than the acute lesion models supplemented with exogenous complement. The result was a less fulminant lesion, more analogous to the subacute astrocytopathy seen in some patients. Future studies could measure complement activity in the serum and determine if complement augmentation leads to more severe astrocyte loss. Additionally, tracking the distribution of the human IgG within the rat ON (for example with fluorescently labeled antibodies) would help clarify how extensively the antibodies penetrate and bind astrocytes—an important consideration for model optimization.

The prominent microglial and macrophage activation we observed in PBS-treated optic nerves underscores the central role of innate immunity in NMOSD pathology [41]. AQP4-IgG triggers astrocytes to release pro-inflammatory cytokines (e.g., IL-1β, TNF-α), which activate microglia and recruit macrophages to the lesion [33,34,35,42,43]. Activated astrocytes also produce complement components, specifically C3 and C3a, which bind to the microglial C3a receptor (C3aR), amplifying the inflammatory cycle [33]. In our model, increased NF-κB, IL-1β, and TNF-α levels in ON tissue corresponded with heavy infiltration of Iba1+ microglia and ED1+ macrophages, along with demyelination (MBP loss). These findings mirror observations in other animal models and ex vivo spinal cord experiments where AQP4-IgG and complement drive robust inflammatory demyelination [44]. The reduction in MBP in PBS eyes to about 35% of the normal amount indicates significant myelin damage was sustained by 4 weeks, and was likely mediated by the membrane attack complexes (MAC) and toxic inflammatory milieu [36]. Our results reinforce that therapies for NMOSD should not only neutralize pathogenic antibodies but should also interrupt the downstream inflammation and glial activation that ultimately cause neurodegeneration [45].

Importantly, this study demonstrates that targeting the ROCK/MYLK4 pathway can effectively protect the ON from immune-mediated damage. ITRI-ES treatment led to better preservation of vision and tissue integrity than the systemic MP regimen. While corticosteroids remain the first-line treatment for acute NMOSD attacks [46,47], their neuroprotective effect is limited—even high-dose IV methylprednisolone often fails to fully prevent relapses or permanent disability [48]. In our model, the MP-treated group (30 mg/kg × 3 days, analogous to moderate dosing) showed only slight improvements in VEP and RGC survival over PBS, underscoring the need for more effective interventions. One factor to consider is that our MP dose was lower than the clinical standard (typically 1000 mg/day IV for several days). However, even at clinical doses, steroids primarily attenuate inflammation and do not directly promote the repair of damaged neurons or glia [49]. By contrast, ITRI-ES appears to confer direct neuroprotection in addition to dampening inflammation.

The benefits of ITRI-ES are consistent with the known effects of ROCK inhibition in the CNS. ROCK1/2 are key regulators of cytoskeletal dynamics, and their inhibition has been shown to enhance neuronal survival, axon regeneration, and blood–brain barrier integrity [50]. In our previous rodent study of anterior ischemic optic neuropathy (rAION), intravitreal ROCK inhibitor (E212) prevented RGC loss and preserved the blood-ON barrier (BONB), leading to improved outcomes [2]. Clinically, intravitreal ROCK inhibitor fasudil has demonstrated improved visual recovery in acute non-arteritic AION [51], highlighting the translational potential of ROCK-targeted therapy for ON diseases. In NMOSD, ROCK activation likely contributes to endothelial permeability and immune cell infiltration across the blood–brain barrier [2,21,23,26]. By stabilizing the barrier and reducing adhesion molecule expression, ROCK inhibition can limit immune cell extravasation into the ON, which is vital for managing acute exacerbations and preventing relapse [20]. Furthermore, ROCK signaling in T cells and macrophages promotes a pro-inflammatory phenotype; its inhibition skews responses toward anti-inflammatory pathways (such as upregulating IL-10 and arginase in macrophages) [24]. Our observation that ITRI-ES increased Arg1 and CD206 levels in the optic nerve indicates a shift toward a healing M2 macrophage/microglia phenotype, which could facilitate tissue repair.

The addition of MYLK4 inhibition may provide further advantages. MYLK4 is a relatively understudied kinase, but it has been implicated in the regulation of astrocyte morphology and possibly migration during injury responses. Astrocytes in NMOSD not only become reactive but can contribute to glial scar formation and chronic inflammation [33,34,42]. We speculate that inhibiting MYLK4 could reduce astrocyte scar formation or excessive migration, thereby mitigating secondary damage [52]. Although we did not directly measure MYLK4 activity in this study, the potent suppression of astrogliosis (GFAP) in ITRI-ES-treated nerves hints that astrocyte reactivity was curtailed, which might be partly due to MYLK4 blockade. Prior in vitro work showed that modulating myosin signaling can affect astrocyte mobility and glial scar components [32]. Thus, dual ROCK/MYLK4 inhibition may simultaneously target multiple cell types—neurons, endothelial cells, microglia/macrophages, and astrocytes—providing a comprehensive therapeutic approach in NMOSD optic neuritis [33].

Our study has several limitations. First, it was based on a single-dose, short-term outcome. We demonstrated significant benefits at 28 days after one intravitreal dose of ITRI-ES, but it is unclear how long these effects will persist or whether additional doses would further improve outcomes. Future studies should explore the optimal dosing frequency and window for intervention (e.g., treating at onset vs. after some delay). We also did not perform direct tracking of the human NMOSD IgG within the rat tissue. Employing labeled antibodies or advanced imaging could verify the spatial distribution of the pathogenic IgG and complement in our model. Second, only male rats were used to minimize variability from hormonal cycles, but future work should include females given the higher prevalence of NMOSD in women. Third, while ITRI-ES pharmacological properties have been described in glaucoma models (showing intraocular bioavailability up to 24 h and selective ROCK1/2-MYLK4 inhibition) [27,28], its pharmacokinetics in NMOSD optic neuritis remain untested. Fourth, in this study the drug was given simultaneously with serum, reflecting a preventive paradigm; whether delayed administration can reverse established pathology requires future evaluation. Fifth, patient serum contains not only AQP4-IgG but also complement and other undefined components. Thus, our model cannot distinguish the relative contribution of IgG versus complement. Future refinements, such as using purified AQP4-IgG with or without complement fractions, would improve mechanistic clarity [53]. Although our model recapitulates humoral immunity, NMOSD pathology also involves T cell-mediated mechanisms. AQP4-specific Th17 cells have been shown to facilitate CNS infiltration and astrocytic injury [54], while rodent immune responses may differ from humans in the degree of T cell involvement [55]. In addition, gliosis and microglial activation were assessed only by Western blot; future studies will incorporate immunolabeling and quantitative cell counts. Lastly, long-term safety, potential effects on systemic immunity, and combinatorial strategies with glucocorticoids or plasma exchange warrant further investigation before translation [56].

## 5. Conclusions

In conclusion, this study provides evidence that dual inhibition of ROCK and MYLK4 can significantly ameliorate ON inflammation and neurodegeneration in an NMOSD model. ITRI-ES treatment preserved visual function, protected RGCs, and reduced hallmark pathological features such as AQP4 loss, demyelination, and gliosis. These findings highlight a novel therapeutic avenue for NMOSD-related optic neuritis, addressing an unmet need for neuroprotective strategies alongside immunosuppression. Given the encouraging preclinical efficacy and safety profile, further development of ROCK/MYLK4 inhibitors—including dose optimization and delivery methods—is warranted. With rigorous clinical testing, such targeted therapies have the potential to improve outcomes and quality of life for patients suffering from NMOSD.

## Data Availability

All data generated or analyzed during this study are included in this published article. Additional datasets are available from the corresponding author on reasonable request.

## Abbreviations

The following abbreviations are used in this manuscript:

AQP4	Aquaporin-4
Arg1	Arginase 1
CD206	Cluster of Differentiation 206
ERG	Electroretinography
GFAP	Glial Fibrillary Acidic Protein
HPF	High-Power Field
Iba1	Ionized Calcium-Binding Adapter Molecule 1
IL-1β	Interleukin-1 Beta
MBP	Myelin Basic Protein
MP	Methylprednisolone
NF-κB	Nuclear Factor kappa-light-chain-enhancer of activated B cells
NMOSD	Neuromyelitis Optica
OCT	Optical Coherence Tomography
ON	Optic Neuritis
PBS	Phosphate-Buffered Saline
RGC	Retinal Ganglion Cell
ROCK	Rho-kinase
TNF-α	Tumor Necrosis Factor Alpha
TUNEL	Terminal deoxynucleotidyl transferase dUTP nick end labeling
